# Precise Diabetic Wound Therapy: PLS Nanospheres Eliminate Senescent Cells via DPP4 Targeting and PARP1 Activation

**DOI:** 10.1002/advs.202104128

**Published:** 2021-11-05

**Authors:** Renliang Zhao, Xiangyun Jin, Ang Li, Bitong Xu, Yifan Shen, Wei Wang, Jinghuan Huang, Yadong Zhang, Xiaolin Li

**Affiliations:** ^1^ Department of Orthopedic Surgery and Shanghai Institute of Microsurgery on Extremities Shanghai Jiao Tong University Affiliated Sixth People's Hospital Shanghai 200233 China; ^2^ Department of Orthopedic Trauma Renji Hospital School of Medicine Shanghai Jiao Tong University Shanghai 200127 P. R. China; ^3^ Department of Spine Center for Orthopaedic Surgery The Third Affiliated Hospital of Southern Medical University Guangzhou 510515 China

**Keywords:** diabetic wound healing, DPP4 receptor, nanospheres, selective targeting, senescence

## Abstract

Diabetic ulcers, a difficult problem faced by clinicians, are strongly associated with an increase in cellular senescence. Few empirical studies have focused on exploring a targeted strategy to cure diabetic wounds by eliminating senescent fibroblasts (SFs) and reducing side effects. In this study, poly‐l‐lysine/sodium alginate (PLS) is modified with talabostat (PT100) and encapsulates a PARP1 plasmid (PARP1@PLS‐PT100) for delivery to target the dipeptidyl peptidase 4 (DPP4) receptor and eliminate SFs. PARP1@PLS‐PT100 releases encapsulated plasmids, displaying high selectivity for SFs over normal fibroblasts by targeting the DPP4 receptor, decreasing senescence‐associated secretory phenotypes (SASPs), and stimulating the secretion of anti‐inflammatory factors. Furthermore, the increased apoptosis of SFs and the disappearance of cellular senescence alleviates SASPs, accelerates re‐epithelialization and collagen deposition, and significantly induces macrophage M2 polarization, which mediates tissue repair and the inflammatory response. This innovative strategy has revealed the previously undefined role of PARP1@PLS‐PT100 in promoting diabetic wound healing, suggesting its therapeutic potential in refractory wound repair.

## Graphical abstract

Poly‐l‐lysine /sodium alginate is modified with talabostat (PT100) and encapsulated with a PARP1 plasmid (PARP1@PLS‐PT100), and nanospheres are developed and applied for targeted drug delivery to the dipeptidyl peptidase 4 (DPP4) receptor to eliminate senescent fibroblasts (SFs). The increased apoptosis of SFs with the disappearance of cellular senescence significantly alleviates inflammation and accelerates re‐epithelialization and collagen deposition in diabetic wounds.

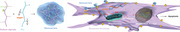

## 1 Introduction

The dramatic increase in the global incidence of type 2 diabetes (T2D) has been accompanied by an increase in the incidence of diabetic foot ulcer (DFU), a leading cause of disability and limb amputation in patients with diabetes.^[^ ^1^, ^2^ ^]^ Pathophysiological oxidative stress and inflammatory disorders frequently result in delayed wound healing.^[^ ^3^, ^4^ ^]^ However, these phenomena of continuous inflammation and oxygen species production in the early stage and later period of wound regeneration are not well understood.^[^ ^5^, ^6^, ^7^, ^8^, ^9^ ^]^ Recently, researchers found that senescent fibroblasts (SFs) accumulate in inflammatory wounds and affect the inflammatory microenvironment.^[^ ^10^, ^11^ ^]^ Pathologically, cellular senescence results in chronic tissue damage and inflammation because of the senescence‐associated secretory phenotype (SASP), which contributes to tumorigenesis, pulmonary fibrosis, atherosclerosis, diabetes, and osteoarthritis.^[^ ^12^, ^13^ ^]^ However, cytokines associated with SASP, including IL‐1*α*, IL‐6, and IFN‐*γ*, induce M1 macrophage polarization,^[^ ^14^ ^]^ thereby activating the inflammatory response in the early stage of impairment.^[^ ^15^ ^]^ Additionally, unrestrained M1 macrophage activation increases the secretion of proinflammatory factors.^[^ ^16^, ^17^ ^]^ Meanwhile, M1 macrophage polarization and continuous inflammation induce the senescence program in fibroblasts in chronic wounds.^[^ ^18^, ^19^ ^]^ Together, these processes comprise a vicious cycle between senescence and inflammation that impairs wound healing.^[^ ^20^ ^]^ Servel studies have contributed to eliminate senescent,^[^ ^21^ ^]^ but few studies have reported an alleviation of the effects of senescence on wound repair by disrupting the vicious cycle. Thus, studies examining the clearance of senescent cells seem to be a feasible strategy for chronic wound therapy.^[^ ^22^ ^]^ However, studies attempting to investigate these strategies have not been completed successfully to date.^[^ ^23^ ^]^ Lamivudine and ruxolitinib modulate the senescence secretome and ameliorate several phenotypes of aging, but these compounds have a complicated mechanism, resulting in an unsatisfactory explanation to prove SASP inhibition.^[^ ^13^, ^24^, ^25^ ^]^ Additionally, the genetic modification of T cells induces chimeric antigen receptors (CARs), which suppress SASPs by targeting mitochondria.^[^ ^26^ ^]^ This pathway was also proposed to mitigate senescence‐induced dysregulation in cells, thereby attenuating the hallmarks of senescence, but the CAR T cell technique does not eliminate defects, including neurotoxicity, cytokine release syndrome (CRS), and several severe side effects.^[^ ^12^, ^27^ ^]^ Various therapies that rely on eliminating senescence for chronic wound healing are less effective.^[^ ^28^ ^]^ Hence, strategies interfering with the antiapoptotic pathway might enable the selective clearance of senescent cells.^[^ ^29^, ^30^, ^31^ ^]^ Highly selective targeted drug delivery systems might target the impaired tissue, achieve better therapeutic efficiency and reduce side effects.^[^ ^32^, ^33^ ^]^ Consequently, the design of a selective targeted drug delivery system loaded with an effective therapeutic factor for chronic wound healing is still a challenge for researchers.^[^ ^4^ ^]^

Mass spectrometry analyses followed by coimmunoprecipitation showed that the surface of SFs exhibits selective dipeptidyl peptidase 4 (DPP4) receptor expression, which was not observed in proliferating human diploid fibroblasts.^[^ ^34^ ^]^ Recent evidence obtained from the transcriptome signature of cellular senescence shows dramatically decreased expression of poly ADP‐ribose polymerase 1 (PARP1) in SFs compared to primary fibroblasts.^[^ ^35^ ^]^ The reduced expression of PARP1 might be related to antiapoptotic effects, immune escape, and other prosurvival mechanisms.^[^ ^36^ ^]^ The role of PARP1, an NAD+/ADP‐ ribosyltransferase, in the cell cycle has been widely studied, and PARP1‐mediated apoptosis is a cell death program.^[^ ^37^ ^]^ PARP1 recruitment and activation lead to the depletion of NAD+, which expedites mitochondrial exhaustion and causes mitochondrial collapse.^[^ ^38^ ^]^ PARP1 characteristically protects normal cells from senescence under physiological conditions and leads to senescent cell apoptosis under pathological conditions to protect against stress.^[^ ^39^ ^]^ Transfection of the PARP1 plasmid regulated the apoptosis of senescent fibroblasts through the direct interruption of the apoptotic pathway by targeting the DPP4 receptor. This strategy is an innovative method in the field of targeted drug delivery systems.^[^ ^40^ ^]^

Diabetic wound ulcer is not only a local wound impairment but also an extensive disorder of the microenvironment characterized by inflammation.^[^ ^41^ ^]^ Several biomaterials have been designed to improve the tissue regeneration in diabetic wound ulcers, such as hydrogels and electrospun nanofibers,^[^ ^42^, ^43^ ^]^ which protect wounds from bacteria and secondary injury but are a less effective treatment for deep tissue. In recent years, nanocarriers and hydrogels have been used to deliver drugs and plasmids and have rapidly progressed.^[^ ^44^ ^]^ In therapeutic plasmid‐based approaches, loading a plasmid into a nanosphere hydrogel enables more efficient transfection.^[^ ^45^ ^]^ A selective drug delivery system targeting senescent cells might essentially reverse disease progression, potentially representing an ideal strategy for eliminating the root cause of diabetic wound recurrence. However, few selective receptor targets present these properties. Here, we describe the design and implementation of PLS‐PT100 nanospheres, which show a higher plasmid‐loading capacity, higher transfection efficiency, and higher sensitivity than liposomes. In this study, PLS nanospheres were modified with PT100 and designed to transfer the PARP1 plasmid, allowing them to target SFs and transfer the PARP1 plasmid into SFs to induce apoptosis. PT100 is a highly selective inhibitor of the DPP4 receptor, which was shown to be abundantly expressed on SFs in a clinical trial and our experiments.^[^ ^46^ ^]^ Treatment with PARP1@PLS‐PT100 nanospheres improved the inflammatory microenvironment and induced SF apoptosis after plasmid transfection. The reduction of the SASP reduced M1 polarization and increased M2 polarization, which benefited wound repair. Meanwhile, the apoptosis of SFs also promoted the differentiation of fibroblasts and wound tissue re‐epithelialization. This study provides a strategy for eliminating SFs and suggests a potential therapeutic intervention for diabetic wound repair.

## 2 Results and Discussion

DFU is a leading cause of disability and limb amputation in patients with diabetes.^[^ ^2^ ^]^ The vicious cycle of senescence and inflammation results in delayed wound healing.^[^ ^22^ ^]^ In our study, we developed a selective targeted delivery system to transport plasmids, which benefits chronic diabetic wound healing. The schematic illustrates therapeutic nanocarriers releasing PARP1 pDNA for wound healing.

### 2.1 Synthesis of PT100‐Modified Sodium Alginate

**Figure 1:**
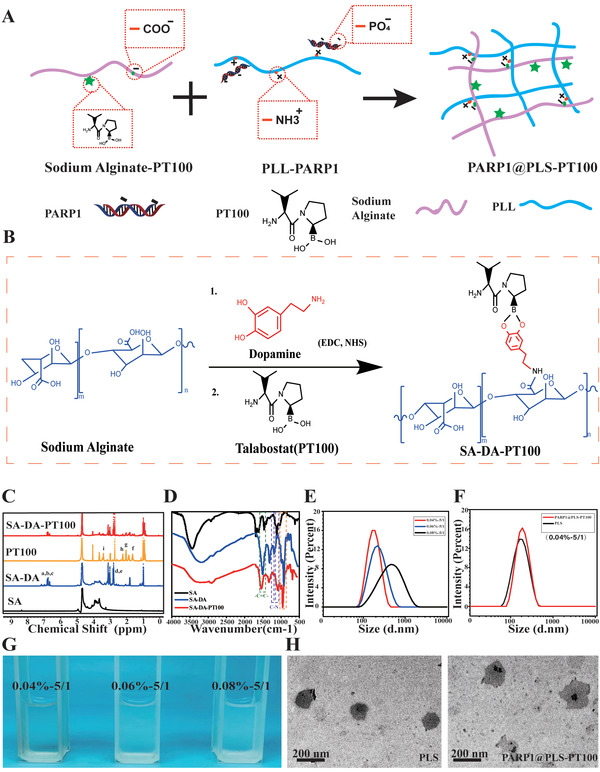
A,B) Schematic illustration of the encapsulation of PARP1@PLS‐PT100 nanospheres; C) NMR analysis confirmed successful sodium alginate scion grafting of PT100; D) FTIR spectrum results showed characteristic bands of SA‐DA‐PT100; G) the morphology of a graded concentration ratio of PLL and SA complex and the E) complex were measured for particle size by NTA. PARP1 encapsulated PLS‐PT100 nanospheres were detected with F) NTA and H)TEM. (*n* = 3 images per group.)

The schematic illustrates the encapsulation of PARP1@PLS‐PT100 nanospheres (**Figure** **1**A,B) ^1^H NMR detection indicated that the catechol group was conjugated to alginate (Figure 1C). Furthermore, ^1H^ NMR detection revealed the successful conjugation of catechol groups due to the presence of peaks for the catechol protons at approximately 7, 2.8, and 3.0 ppm. Moreover, the ^1^H NMR analysis revealed distinct peaks at 3.5 ppm (‐B‐C‐) and from 1.9–2.1 ppm (CH_2_), which were attributed to PT100 (Figure 1C). On the other hand, the FTIR spectrum of alginate‐dopamine showed characteristic bands corresponding to aromatic C═C bonds (1517 cm^–1^) and stretching bands at 1080 and 1183 cm^–1^ corresponding to C—N. The peak at 1365 cm^–1^ was related to the presence of the boric acid group of PT100 (Figure 1D). These results indicated the successful conjugation of the small molecule PT100.

### 2.2 Characterization of PARP1‐Loaded PLS‐PT100 Complex Particles

This study aims to synthesize polyelectrolyte complex nanoparticles from sodium alginate and cationic PLL for plasmid delivery. The formation of PLS nanoparticles depends on the concentration and mixing ratio of the solution. Alg/PLL polyelectrolyte complex nanoparticles were prepared by simply mixing PLL and Alg at different masses. The PLS compound was evident from the turbid appearance when the two solutions were mixed (Figure 1G). In subsequent experiments, poly‐l‐lysine was added to the alginate solution to determine the concentration range appropriate for particle formation. The effect of the increasing solid content on particle size was evaluated at a fixed alginate/PLL ratio of 5:1 (w/w). Particles were obtained with a Na‐alginate solid content ranging from 0.04 to 0.08%, and higher concentrations led to solution‐like systems with larger aggregates. Actively targeted small particles present advantages over larger passively targeted nanospheres due to the enhanced permeability and retention (EPR) effect. We obtained particles of approximately 100–200 nm in size to facilitate cellular phagocytosis; therefore, we chose a formula with a constant alginate/PLL ratio of 5:1 (w/w) and a total solid content of 0.04% for subsequent studies, and the spherical morphology was maintained even after drug loading (Figure 1E,H). After modification with the small molecule PT100 and plasmid DNA (pDNA), no obvious change in particle size was detected (Figure 1F). The transmission electron microscopy (TEM) analysis showed that both of the complexes formed self‐assembled spheres when dispersed in water (Figure 1H).

### 2.3 The PARP1 Plasmid Was Efficiently Transfected into SFs by PLS‐PT100 Nanospheres

**Figure 2:**
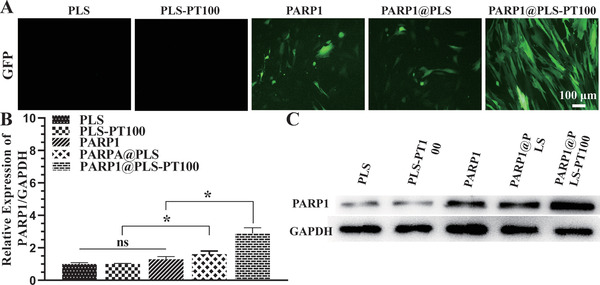
A) GFP expression was indicated by green fluorescence in SFs after transfecting with liposomes, PLS, PLS@PT100. B) The relative PARP1 gene expression in SFs with the indicated treatment and the C) expression of the protein. (*n* = 3 per group; * *p* < 0.05, ns, *p* > 0.05.)

The application of liposomes to deliver plasmid DNA (pDNA) is limited because of variations in transfection efficiency,^[^ ^47^ ^]^ and microcapsules fabricated from nanospheres were designed as delivery vehicles for engineered bioactive peptides and pDNA. PLS nanospheres, which exhibit excellent biocompatibility and efficient delivery, show significant potential for clinical diabetic wound therapy. Nucleic acid electrophoresis was used to determine the proper loading amounts of nanospheres to pDNA. PLS‐PT100 nanospheres (400 µg) were incubated with various concentrations of the PARP1 plasmid, and Lipofectamine solutions were used to transfect the plasmid as a control. In this case, 1 represents the marker, 2 represents Lipofectamine, and 3–8 represent a series of pDNA concentrations (50, 40, 30, 20, 10, and 5 µg). The loading amount was obtained from the electrophoresis result (Figure S1A, Supporting Information), and 400 µg of nanospheres containing 40 µg of plasmid were considered an appropriate loading amount. The PARP1 plasmid was transfected into SFs using Lipofectamine or PLS‐PT100 nanospheres, which were observed by detecting GFP signals. GFP fluorescence images were collected 24 h after transfection of the GFP‐PARP1 plasmid. Transfection efficiency was evaluated by the expression of GFP protein in SFs after transfections. More fluorescence was observed in the PARP1@PLS‐PT100 group, and the fluorescence intensity was the highest compared with the liposomes and PLS nanocarriers (**Figure** **2**A). Furthermore, the transfection efficiency was determined by performing RT‐PCR and Western blotting (WB), and the results showed that significantly more PARP1 mRNA was amplified in the PLS‐PT100 group than in the Lipofectamine group (Figure 2B). The Western blot results indicated a similar trend; more PARP1 plasmid was transfected into SFs by PARP1@PT100 than Lipofectamine (Figure 2C). More PARP1 plasmid was released from the nanospheres than liposomes and led to increased expression of PARP1 in SFs in this study. Overall, our research successfully reported a selective delivery system that resolved the inefficient transfection by liposomes.

### 2.4 Elimination of SFs by PARP1@PLS‐PT100 Alleviated Inflammation and Restored the Physiological Function of HFF‐1 Cells In Vitro

**Figure 3:**
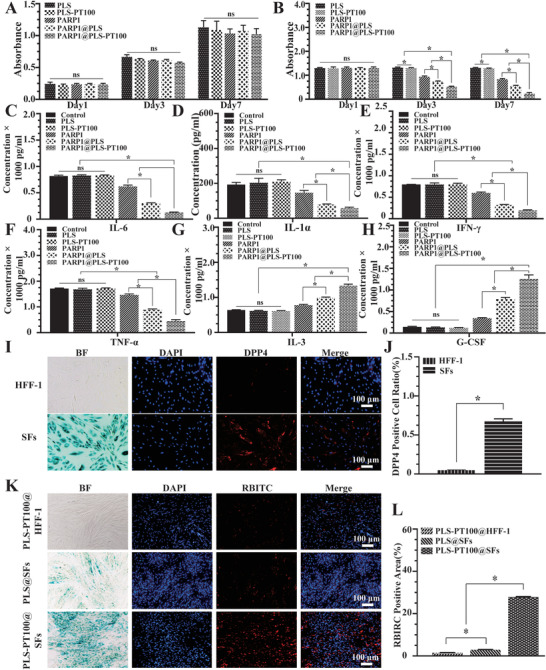
A) Proliferating human foreskin fibroblasts (HFF‐1) and B) senescent fibroblasts (SFs) were incubated with a series of nanospheres. The expression of SASPs: C) IL‐6; D: IL‐1*α*; E: IFN‐*γ*; F) TNF‐*α*) and anti‐inflammatory factors: G) IL‐3; H) G‐CSF was detected in HFF‐1 cells, which were incubated with the supernatant collected from SFs after treatment with PBS, PLS, PLS‐PT100, PARP1, PARP1@PLS, PARP1@PLS‐PT100 nanocarriers. Selective expression of DPP4 receptor in SFs compared with I) normal HFF‐1, J) quantitative evaluation of the DPP4 positive cell ratio. K) The immunofluorescence staining images indicated that PT100 modified nanospheres could target SFs, and L) the quantitative results were calculated. (*n* = 3 per group; * *p* < 0.05, ns, *p* > 0.05).

Cell viability and physiological functions were measured after the treatment with nanospheres. The viability of HFF‐1 cells and SFs treated with nanospheres was detected after 1, 3, and 5 days. The PARP1@PLS‐PT100 nanospheres had no significant effect on the proliferation of HFF‐1 cells at each time point (**Figure** **3**A). In contrast, a different effect on the growth of the SFs was observed in the PLS group, and the number of SFs decreased upon PARP1@PLS, PARP1, and PARP1@PLS‐PT100 nanosphere treatment for 3 and 7 days. Furthermore, the PARP1@PLS‐PT100 group contained significantly fewer SFs than the PARP1@PLS group (Figure 3B), and the results indicated that nanospheres induced the death of SFs due to the transfection of plasmids rather than cytotoxicity. The SASP causes disordered inflammation and senescence progression.^[^ ^48^ ^]^ SFs (5.6 × 10^4^ cells cm^−2^) were seeded in culture plates, and incubated for 24 h, then the medium was changed to fresh complete medium containing 200 µg of nanospheres, equal valume of PBS were added as control group. 48 h later, the supernatants were collected and cultured with HFF‐1 cells for 1 day, and then the levels of SASPs and inflammatory factors were detected with ELISA kits. The expression levels of IL‐6 (Figure 3C), IL‐1*α* (Figure 3D), IFN‐*γ* (Figure 3E), and TNF‐*α* (Figure 3F) were significantly decreased in the PARP1@PLS‐PT100 group compared with the PLS group. Moreover, the expression levels of IL‐3 (Figure 3G) and G‐CSF (Figure 3H) were higher than those in the PARP1@PLS group. Additionally, no significant difference in the expression of IFN‐*γ*, IL‐1*α*, IL‐6, TNF‐*α*, IL‐13, and G‐CSF was observed in the PLS‐PT100 group compared with the PLS group and control group. Nanospheres alleviated inflammation and increased anti‐inflammatory factors by transfecting plasmids. HFF‐1 cells and SFs were distinguished by senescence‐related *β*‐galactosidase staining (Figure 3I), and the DPP4 receptor‐positive cell ratio of the SF group was higher than that of the HFF‐1 group (Figure 3I,J). Thus, the DPP4 receptor (red) is selectively expressed on SFs rather than HFF‐1 cells. The targeting ability of microcapsule nanocarriers relies on the interaction of nanospheres and the cell surface.^[^ ^49^ ^]^ HFF‐1 cells and SFs were used to evaluate the transfection efficiency of the nanospheres labeled with rhodamine B isothiocyanate (RBITC). PLS nanospheres were added in to SFs (PLS@SFs), PLS‐PT100 nanospheres were added into HFF‐1 (PLS‐PT100@HFF‐1), and SFs (PLS‐PT100@SFs), respectively. More red fluorescence was detected after 24 hours of incubation in the PLS‐PT100@SFs group than in the PLS@SFs group and PLS‐PT100@ HFF‐1 group (Figure 3L), which confirmed that the PLS‐PT100 nanospheres were an efficient and stable delivery strategy.

**Figure 4:**
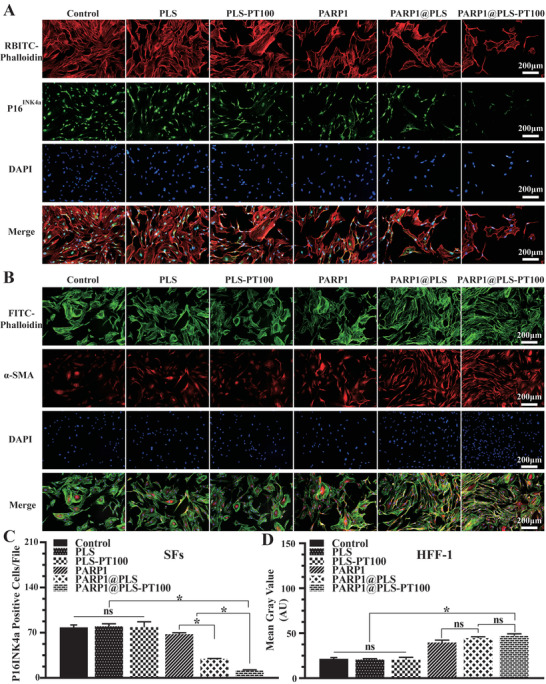
A) The expression of P16^INK4a^ was measured ( and the C) number P16^INK4a^ of positive cells was also calculated. B) The immunofluorescence staining images of *α*‐SMA on HFF‐1 cells with the treatment of SFs’ supernatant and the D) mean fluorescence intensity (MFI) were calculated. (*n* = 3 per group; * *p* < 0.05, ns, *p* > 0.05).

P16^INK4a^ is an important biomarker of cell cycle arrest and senescence.^[^ ^50^, ^51^ ^]^ Senescence was attenuated after the transfection of the PARP1 plasmid. SFs were seeded in 24‐well plates at a density of 5.6 × 10^4^ SFs cells per well and then stained after 3 days of treatment with the nanospheres or PBS (**Figure** **4**A). The number of P16^INK4a^‐positive SFs was significantly decreased in the PARP1@PLS group, PARP1@plasmid group, and PARP1@PLS‐PT100 group compared with the PLS group and control group.(Figure 4C). These results obviously indicated the relief of senescence. Cell senescence leads to altered differentiation of fibroblasts and impairs wound regeneration. Myofibroblasts contribute substantially to tissue regeneration, and the expression of *α*‐smooth muscle actin (*α*‐SMA) is a characteristic of activated fibroblasts.^[^ ^52^, ^53^ ^]^ For this experiment, 2.8 × 10^4^ HFF‐1 cells cm^−2^ were cultured with supernatant collected from the SFs after treatment with nanospheres for 24 hours. *α*‐SMA expression was assayed and was significantly decreased in the PLS group compared with the PARP1@PLS‐PT100 group (Figure 4B). In the PARP1@PLS‐PT100 group, *α*‐SMA was expressed at significantly higher levels than that in the control group, PLS group, and PLS‐PT100 group (Figure 4D), and its expression was not significantly different between the PARP1@PLS‐PT100 and PARP1@PLS groups. The preceding stages of the study revealed the effect of PARP1@PLS‐PT100 nanospheres on restoring the differentiation of fibroblasts by alleviating inflammatory stimulation. With the decrease in the number of SFs, the secretion of proinflammatory factors was decreased, and the anti‐inflammatory effects were increased in vitro. The elimination of senescence and inflammation promotes the differentiation of myofibroblasts, which contributes substantially to wound healing.

### 2.5 The PARP1 Plasmid Promoted the Apoptosis of SFs and Alleviated the Senescent Phenotype

**Figure 5:**
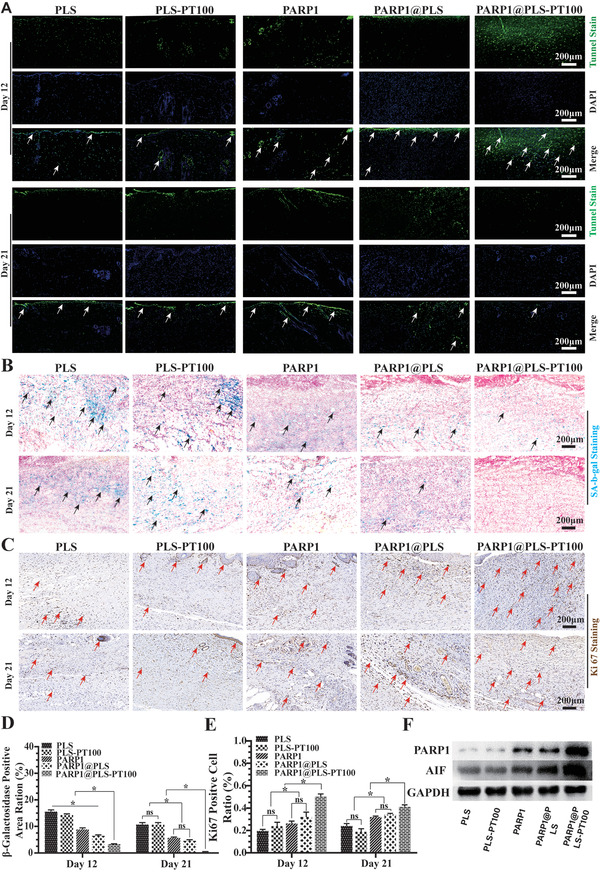
A) TUNEL fluorescence staining of wound tissues (. Staining of senescence‐associated B) b‐galactosidase (SA‐b‐gal) and the D) SA‐b‐gal‐positive area were calculated. C) Immunohistochemistry staining of Ki67 in wound sections at day 12 and day 21 after surgery and E) quantitative evaluation of the Ki67‐positive cell ratio. F) The expression of PARP1 and AIF in wound tissue on day 12 after surgery. (*n* = 3 per group; * *p* < 0.05, ns, *p* > 0.05).

Several studies have shown that obliterating senescent cells may alleviate senescence‐induced dysfunction and inflammation to reduce the burden on normal fibroblasts.^[^ ^54^, ^55^ ^]^ Several studies have focused on targeting SFs, and existing therapies, such as CAR T cell therapy, are accompanied by rare side effects. Typically, SFs displaying increased senescence and reduced apoptosis are especially resistant to immune cells.^[^ ^6^ ^]^ Selective targeting and elimination of SFs appears necessary and important. In order to evaluated the effection of nanospheres on SFs in the diabetic wound, wound tissue were collected for staining. TUNEL staining is an important phenotype of apoptosis, which was evaluated with immunofluorescence staining, and positive TUNEL staining was visualized using fluorescein isothiocyanate (FITC) (**Figure** **5**A). The largest area of TUNEL‐positive staining was observed in the PARP1@PLS‐PT100 group on day 12; additionally, the number of apoptotic cells in the PARP1@PLS‐PT100 group was lower on day 21 than on day 12 after surgery (Figure S1B, Supporting Information). Transfecting PARP1 plasmid into SFs promoted apoptosis of SFs and alleviated the senescent phenotype, relieving the wound healing problem in diabetic wounds and other refractory chronic wounds.

Diabetic wound healing is an urgent clinical problem, and few effective methods are available to protect against the incidence of diabetic ulcers and their recurrence.^[^ ^56^ ^]^ Various treatments for diabetic ulcer healing have been studied, but the disrupted microenvironment is difficult to resolve through revascularization and antibacterial agents.^[^ ^57^, ^58^ ^]^ Here, we found that SFs accumulate in diabetic wounds and might be a potential target for restoring the self‐repair capacity of wound tissue.^[^ ^59^, ^60^ ^]^ Senescence‐related *β*‐galactosidase staining of diabetic wound sections was performed, and the expression of the senescence‐related *β*‐galactosidase protein decreased following PARP1@PLS, PARP1, and PARP1@PLS‐PT100 treatment compared with the PLS group. No significant difference in the expression of senescence‐related *β*‐galactosidase was detected on days 12 and 21 between the PLS and PLS‐PT100 groups (Figure 5B). In addition, the results of senescence‐related *β*‐galactosidase staining confirmed the elimination of senescent cells from the wound. Among the groups, senescence was relieved most significantly in the PARP1@PLS‐PT100 group (Figure 5D). Ki67 represents the recovery of impaired tissue when senescence has been eliminated. Immunohistochemical staining of wounded skin for Ki67 revealed more Ki67‐positive cells in the PARP1@PLS, PARP1, and PARP1@PLS‐PT100 groups than in the PLS group, especially in the PARP1@PLS‐PT100 group (Figure 5C). Furthermore, the quantification of Ki67‐positive cells on days 12 and 21 showed more positive cells in the PARP1, PARP1@PLS, and PARP1@PLS‐PT100 groups than in the PLS group (Figure 5E), indicating that senescence may impair the wound regeneration and that PARP1@PLS‐PT100 nanospheres contributed significantly to relieving the inhibition mediated by the elimination of SFs. Additionally, the flow cytometry analysis of wound tissue also produced a similar result (Figure S1C,D, Supporting Information).

The PARP1 gene is related to DNA damage and apoptosis.^[^ ^61^ ^]^ Senescence may protect cells with DNA damage from neoplasia because low PARP1 expression in senescent cells might be associated with the inhibition of apoptosis and immune escape.^[^ ^39^, ^62^ ^]^ Increased PARP1 activity might promote cell death and ATP depletion,^[^ ^37^ ^]^ and high PARP1 expression promotes apoptosis‐inducing factor (AIF) activation and induces the apoptosis of senescent cells.^[^ ^12^, ^37^, ^63^ ^]^ In our study, when the PARP1 plasmid was transfected into senescent cells, increased apoptosis was observed, and the SASP was also alleviated. In addition, normal fibroblasts proliferated normally after transfection. The WB results revealed that overexpressed PARP1 induced the expression of AIF and SF apoptosis (Figure 5F). Thus, PARP1 is a potential target to regulate the apoptosis of SFs and protect normal fibroblasts from senescence. Above results revealed that PARP1@PLS‐PT100 nanospheres could efficiently eliminate SFs, and the Ki67 positive cells suggested the beginning of the regeneration after relif of senescence, thus, more efforts were to probe the mechanism via detection of immune cells and fibroblasts.

### 2.6 The Elimination of SFs Regulated Inflammation and Promoted Wound Healing

SASPs are severe phenotypes associated with the secretion of IL‐6 and TNF‐*α* from senescent cells.^[^ ^64^ ^]^ SASPs are characterized by cell growth arrest and resistance to apoptosis, a well‐known senescent phenotype.^[^ ^65^, ^66^ ^]^ The progression of senescence causes the development of inflammation in diabetic wound healing, and continuous inflammation contributes significantly to senescence induction.^[^ ^67^ ^]^

**Figure 6:**
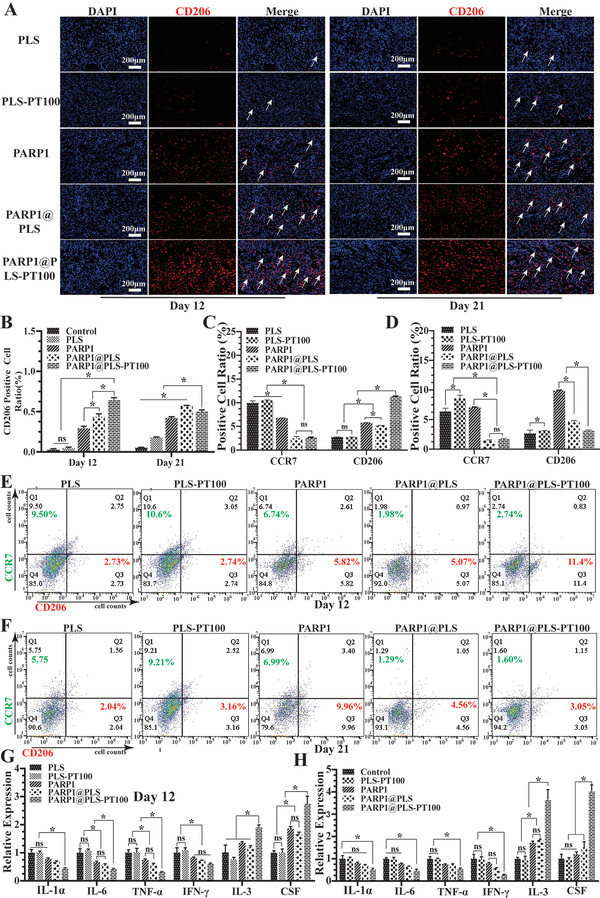
A) Immunofluorescence staining of CD206 and B) quantitative evaluation of CD206‐positive cells. E,F) Flow cytometry of wound tissue at days 12 and 21 after surgery, and the C,D) analysis of flow cytometry were calculated. The relative SASP (IL‐1*α*, IL‐6, TNF‐*α*, and IFN‐*γ*) and anti‐inflammatory factor (IL‐3, CSF) gene expression in rat wound tissues at G) day 12 and H) day 21, (*n* = 3 per group; * *p* < 0.05, ns, *p* > 0.05).

The vicious cycle between senescence and inflammation causes pathological wound healing.^[^ ^20^ ^]^ Thus, elimination of senescence is important to regulate the inflammatory microenvironment and maintain tissue self‐repair integrity. The mannose receptor CD206 is a C‐type lectin that is primarily found on the surface of macrophages and human fibroblasts; it contributes substantially to modulating inflammation.^[^ ^53^ ^]^ CD206 immunofluorescence staining was performed to evaluate the level of inflammation on days 12 and 21, and the results indicated that CD206 was expressed at a low level in the PLS group on day 12 but increased with the repair of the wound on day 21. Furthermore, CD206 expression did not significantly differ between the PLS and PLS‐PT100 groups. Upon PARP1@PLS, PARP1, and PARP1@PLS‐PT100 nanosphere treatment, CD206 expression was significantly increased compared with that in the other groups, and the largest CD206‐positive area was detected on day 12 in the PARP1@PLS‐PT100 group. On day 21 after surgery, the expression of CD206 in the PARP1@PLS‐PT100 group was slightly lower than that on day 12 (**Figure** **6**A), and the ratio of CD206‐positive cells on day 12 was the highest in the PARP1@PLS‐PT100 group (Figure 6B). The wound tissues were detected using flow cytometry to assess macrophage polarization, and the results confirmed that PARP1@PLS‐PT100 nanospheres reduced the polarization of M1 macrophages and promoted M2 macrophage polarization on day 12 because of lower levels of CCR7 and higher levels of CD206 (Figure 6C). After complete healing, the levels of M1 and M2 macrophages were lower in the PARP1@PLS‐PT100 group (Figure 6D–F). Furthermore, RT‐PCR analysis of the wound tissue on days 12 and 21 confirmed higher expression of inflammatory factors (IFN‐*γ*, TNF‐*α*, IL‐6, and IL‐1) in the PLS and PLS‐PT100 groups than in the other three groups. Anti‐inflammatory factors (IL‐3 and CSF) were expressed at higher levels on days 12 and 21 after surgery in the PARP1@PLS‐PT100 group than in the PLS group (Figure 6G,H). The results confirmed that a continuous inflammatory microenvironment had been restored with the delivery of pDNA by the nanospheres, and the reduction in senescence promoted M2 macrophage polarization and wound regeneration.

Diabetic wound tissue regeneration was accelerated by the elimination of SFs, and a reduction in SASPs improved the inflammatory microenvironment. Injection of the nanospheres induced the apoptosis of SFs and restored the proliferation of fibroblasts and immune cells. Overall, previous studies indicated that overactivation of PARP‐1 activated AIF, resulting in the apoptosis of senescent cells, consistent with several previous studies.^[^ ^68^ ^]^ The vicious cycle between senescence and inflammation ended with the clearance of senescent cells by nanocarriers.

### 2.7 Eliminating SF Accumulation Promoted Diabetic Wound Healing In Vivo

**Figure 7:**
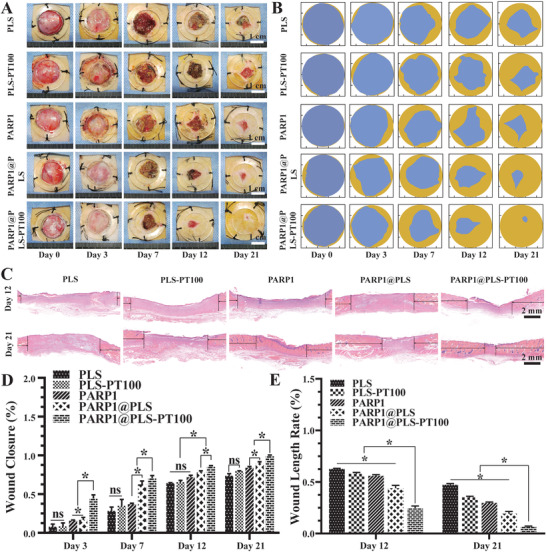
A) Gross photographs of wound closure and B) simulation plots of wound closure. C) H&E staining of the wound indicated the healing situation on days 12 and 21. The quantitative analysis of D) wound closureand E) H&E staining. (*n* = 3 per group; * *p* < 0.05, ns, *p* > 0.05.)

Normally, clearance of senescent cells allows the regeneration of damaged tissues.^[^ ^6^, ^54^, ^55^ ^]^ Wound tissue was collected on days 12 and 21 after surgery. A digital camera was used to collect representative images for the direct visualization of wound closure at the top of the wound on days 0, 3, 7, 12, and 21 after surgery (**Figure** **7**A). A schematic showing wound closure progression based on the images is provided (Figure 7B). The wound closure area was calculated, and wound healing was significantly enhanced by the PARP1@PLS‐PT100 nanospheres compared with the other groups. Wound healing did not differ significantly between the PLS‐PT100 and PLS groups (Figure 7D). H&E staining illustrated the process of re‐epithelialization of the wound tissue in the PLS, PLS‐PT100, PARP1@PLS, PARP1, and PARP1@PLS‐PT100 groups (Figure 7C); the wound healing rate was increased in the PARP1@PLS‐PT100 group compared with the PLS group (Figure 7E). The wound length was calculated and a similar result was obtained, consistent with the schematic showing the rate of wound closure. Collectively, tissue re‐epithelialization was restored by clearance of the SFs.

### 2.8 The Elimination of SFs Promoted Collagen Deposition

**Figure 8:**
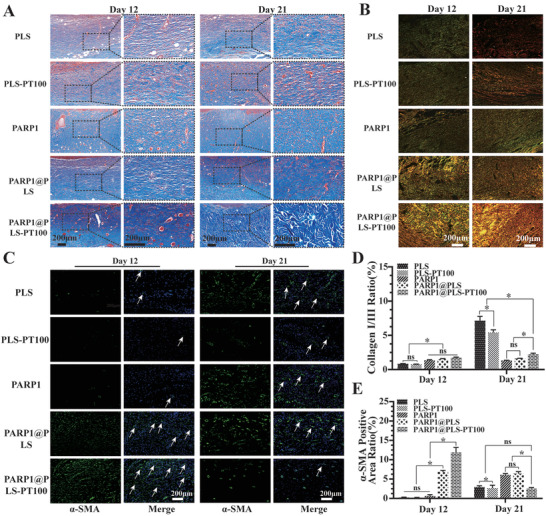
A) Masson staining and B) Sirius red staining indicated collagen deposition and alignment. D) The analysis of Sirius red staining indicated the ratio of collagen I (yellow) / collagen III (green). C) The immunofluorescence staining images of *α*‐SMA and the E) quantitative analysis of *α*‐SMA positive area rate. (*n* = 3 per group; * *p* < 0.05, ns, *p* > 0.05.)

The accumulation of collagen benefits wound healing, and the proportion and alignment of collagen significantly contribute to differentiating normal wound healing and pathological wound healing.^[^ ^59^ ^]^ Therefore, Masson's trichrome staining and Sirius red staining were used to evaluate collagen deposition and alignment, respectively. Masson's trichrome staining indicated collagen deposition on the callus on days 12 and 21 after surgery (**Figure** **8**A). The PARP1@PLS‐PT100 group showed significantly more collagen accumulation than the PLS group. The alignment of collagen bundles was most similar to normal skin in the PARP1@PLS‐PT100 group, and the collagen structure in the PLS group was similar to refractory diabetic wounds. Cell cycle arrest is the main feature of SFs, and inflammation and abnormal proliferation cause pathological diabetic wound healing because of SFs. We confirmed the clearance of SFs after treatment with PARP1@PLS‐PT100. As a complement, Sirius red staining appropriately indicated the alignment of collagen bundles (Figure 8B), and the collagen alignment and collagen I/III ratio were similar to those of normal skin upon treatment with the PARP1@PLS‐PT100 nanospheres (Figure 8D). Furthermore, An ELISA kit were used to quantitate the collagen I and III, its results revealed that the quantitation of collagen I and III in the PARP1@PLS‐PT100 is highest than other groups at day 12, its expression decline in the day 21(Figure S2A–C, Supporting Information). *α*‐SMA expression is also necessary for the formation of myofibroblasts, which can contract the edges of the wound and result in the transformation of fibroblasts.^[^ ^44^ ^]^ Plastic loops were sutured around the rat wound to inhibit contraction of the wound edge and to allow wound repair through granulation and re‐epithelialization, mimicking the repair and remodeling of chronic wounds in humans. The expression of *α*‐SMA confirmed the contraction of the wound edges and indirectly indicated growth of the tissue surrounding the wounds at different time points. FITC fluorescence was used to indicate *α*‐SMA expression, and the PARP1@PLS‐PT100 group showed the largest fluorescence area and ratio on day 12 (Figure 8C). *α*‐SMA expression was increased on day 21 in the PLS, PLS‐PT100, PARP1@PLS, and PARP1 groups, and the results indicated that wound repair was ongoing. A decrease in *α*‐SMA expression was observed on day 21 compared to day 12 in the PARP1@PLS‐PT100 group (Figure 8E), indicating that wound repair was accomplished. Therefore, only a small amount of *α*‐SMA expressed on the blood vessels was observed after complete repair of the wound. Additionally, a flow cytometry analysis of the wound tissue was performed on days 12 and 21, and the results indicated that the expression of fibroblast‐specific protein‐1 (FSP‐1) was increased on day 12 in the PARP1@PLS, PARP1 groups, and PARP1@PLS‐PT100 group compared with the PLS and PLS‐PT100 groups. Moreover, 21 days after wounding, the number of fibroblasts also decreased in the PARP1@PLS‐PT100 group (Figure S1E,F, Supporting Information).

Senescence is a special cellular state against death.^[^ ^69^ ^]^ Recently, interest in the therapeutic targeting of senescence for promoting tissue regeneration and improving senescence‐related disease has increased.^[^ ^70^, ^71^ ^]^ Coimmunoprecipitation experiments revealed that DPP4 was selectively expressed in SFs, and PT100‐modified nanospheres might critically bind to SFs through the DPP4 receptor. Here, we established a full‐thickness cutaneous diabetic wound defect model in which PARP1@PLS‐PT100 nanospheres were injected around the wound. In vivo results showed an increased capacity to repair the tissue in diabetic wounds. As expected, the PARP1 plasmid was delivered into SFs and resulted in a significant decrease in SASPs. Afterward, wound healing was accelerated, as indicated by significant increases in wound re‐epithelialization, and collagen deposition, and immunoregulation. In conclusion, our research has revealed a novel transfection system and selective targeting sites for SFs, and the strategy presents potential for the therapy of diabetic wounds. Because of its ability to selectively target SFs, this system can deliver proteins and pDNA to treat specific senescence‐associated diseases. Therefore, the present strategy might have broader implications in the treatment or prevention of other senescence‐associated diseases, such as age‐related osteoporosis and degenerative arthritis.

## 3 Conclusions

Methods to manage chronic refractory wounds in patients remain a long‐standing puzzle.^[^ ^72^ ^]^ Here, we designed an injectable agent based on the targeted delivery of a PARP1 plasmid for the treatment of this condition. This delivery system facilitated targeting of the therapeutic plasmid to SFs and showed a high capacity and efficient loading of the PLS plasmid as a therapeutic and curative strategy. The continuous inflammatory microenvironment and senescence caused by SFs led to reduced proliferation and disordered re‐epithelialization. The PARP1 plasmid was selectively transfected into SFs by targeting DPP4 receptors and effectively reduced SASPs, which promoted wound healing with a decrease in the number of M1 macrophages and an increase in the number of M2 macrophages by disrupting the vicious cycle between inflammation and senescence. The injectable nanospheres could easily be customized to carry various plasmids for the treatment of deep and enclosed wounds in patients with a wide array of clinical conditions, such as diabetic retinopathy and diabetic neuropathy, via a minimally invasive procedure. PARP1@PLS‐PT100 nanospheres mitigate diabetes‐induced dysregulation in cells, thereby attenuating senescence and restoring tissue repair.

## 4 Experimental Section

### Synthesis of PT100‐Modified Alginate

**Scheme 1:**
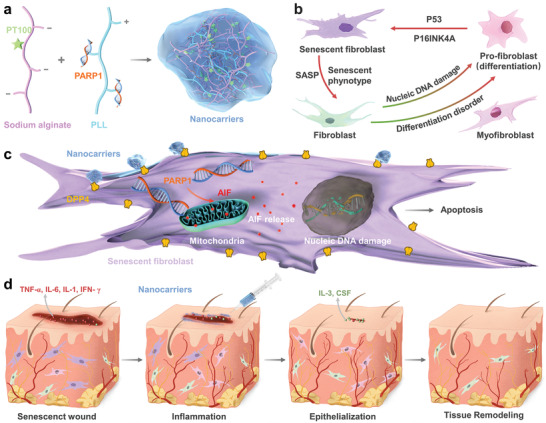
Schematic illustration of therapeutic nanocarriers releasing PARP1 pDNA for wound healing. a) Preparation of PARP1@PLS‐PT100 nanocarriers. b) senescence is the significant source of inflammation and persistent continuing inflammatory microenvironment, and inflammation accelerates the progression of senescence. c) Sustained release of pDNA with the injection of nanospheres promoted the expression of AIF and its release and leading to the senescent fibroblast apoptosis d) Regeneration of wound Senescenct wound healing process tissue by the composited with PARP1@PLS‐PT100 therapeutic nanospheres.

The synthesis of sodium alginate‐PT100 was performed in two steps, as illustrated in **Scheme** **1**. First, sodium alginate‐dopamine was synthesized. Sodium alginate (1.0 g) was dissolved in distilled water. Then, EDC (191.7 mg) and NHS (115.09 mg) were added to this solution. The reaction mixture was stirred at room temperature for 60 min to fully activate the carboxylic groups on alginate molecules. Then, 189.6 mg of dopamine were added to the aforementioned mixture and stirred for 12 h at room temperature under N_2_ protection. The product was dialyzed in water in the dark, followed by lyophilization. The as‐synthesized dopamine‐modified alginate (85.0 mg) was then reacted with the small molecule PT‐100 (30 mg) in PBS to synthesize PT‐100‐modified alginates.

### Preparation of PARP1‐Loaded PT‐100‐Modified Alg/PLL Polyelectrolyte Nanoparticles

PLL/sodium alginate polyelectrolyte nanoparticles were prepared through reaction between carboxyl and amino groups via electrostatic interactions. Solutions of sodium alginate (Alg) and cationic PLL (Alg/PLL = 5:1) were prepared in distilled water. The polyelectrolyte complex was prepared by adding an aqueous solution of PLL to Alg at room temperature with vigorous vortexing for 5 min. Polyelectrolyte complexes of different compositions were prepared by varying the content of Alg (0.04%‐0.08% wt.%). PARP1‐loaded PT100‐modified Alg/PLL polyelectrolyte nanoparticles were prepared using a similar method as mentioned above, but alginate and PLL were converted into PT100‐modified alginate and PARP1‐loaded PLL.

### Characterization of PARP1@PLS‐PT100 Nanocarriers

Syntheses of alginate‐dopamine and alginate‐dopamine‐PT‐100 were confirmed by measuring ^1^H nuclear magnetic resonance (NMR) spectra on a 400 MHz NMR spectrometer and Fourier transform infrared spectrometer (FTIR, Nicolet 6700). The hydrodynamic diameter of NPs was determined in an aqueous solution using dynamic light scattering (Zetasizer Nano ZS) and transmission electron microscopy (TEM, JEM‐2100).

### Cells and Reagents

The human foreskin fibroblast cell line (HFF‐1 cells) was purchased from the Chinese Academy of Sciences. For senescence induction, 10 µg mL^−1^ mitomycin C (MMC) was added to confluent HFF‐1 cells and incubated for 3 hours. The cells were rinsed with PBS and then incubated for 48 hours. SFs were confirmed by *β*‐galactosidase (SA‐*β*‐Gal) staining. HFF‐1 cells and SFs were incubated with high glucose complete DMEM supplemented with 10% FBS (Sigma) and 1% penicillin‐streptomycin at 37°C in an atmosphere containing 5% CO_2_.

### Identification of the SFs

SFs (2.8×10^4^ cells cm^−2^) were seeded in 24‐well plates. After treatment with nanospheres for 24 hours, the medium was removed, and the plates were rinsed three times with PBS. SA‐*β*‐Gal fixation solutions (Solarbio, Beijing, China) were added to the plate to ensure that the solution covered the surface completely. The plates were incubated overnight at 37°C in the dark without CO_2_. The solution was removed completely and the plates were washed with PBS. Cells in each well were observed and photographed using a Leica bright‐field microscope, and SA‐*β*‐Gal‐positive cells were calculated.

### Cell Viability of Primary Fibroblast and SFs

The viability of HFF‐1 cells and SFs was detected with a commercial cell counting kit‐8 (CCK‐8) assay kit (Dojindo Laboratories, Kumamoto, Japan). HFF‐1 cells and SFs were seeded at densities of 3 × 10^3^ and 3 × 10^4^ cells cm^−2^, respectively, in clear 96‐well plates. The cells were incubated with dilutions of various nanospheres in 10% FBS‐containing complete medium, and the medium was changed every two days. Viability was assessed 1, 3, and 5 days by incubating the cells with 100 µl of 10% CCK‐8 detection solution for 1.5 hours at 37 °C. The absorbance was detected at 450 nm with a multidetection microplate reader at the indicated time points (BioTek, USA).

### Nanoparticles Targeting to the SFs

HFF‐1 cells and SFs(5.6 × 10^4^ cells per well) were cultured in 24‐well plates. Cells were attached to plates 24 hours after incubation, and 500 µL of culture medium were replaced with 100 µg mL^−1^ rhodamine B isothiocyanate (RBITC)‐labeled nanosphere‐containing medium for 24 hours. Before the staining procedure, the medium was removed from the plates, and the plates were rinsed with PBS three times. Fixation was performed for 30 min at room temperature (RT) with 4% paraformaldehyde solution. Briefly, 200 µL of SA‐*β*‐Gal fixation solution (Solarbio, Beijing, China) were added to the plate, which was incubated in the dark for 12 h without CO_2_. Fluorescence and bright‐field images were observed and captured using a Leica fluorescence microscope (Leica).

### Rat Surgical Procedure

Sprague‐Dawley (SD) rats (6–8 weeks old) were fed at the Experimental Animal Center of Shanghai Jiaotong University Affiliated Sixth People's Hospital (Shanghai, China). All animal experiments were approved by the ethics committee of the Shanghai Jiaotong University Affiliated Sixth People's Hospital (Shanghai, China). A genetically diabetic rat model that has been well established as a model for chronic wound healing was prepared as follows: SD rats were fed a high‐fat (HF) diet for 2 weeks before streptozotocin (STZ) (100 mg kg^−1^, i.p.) injection, and rats continued to be fed a HF diet throughout the experimental period.^[^ ^13^ ^]^ After inducing the type 2 diabetes model, blood glucose levels were measured 2 weeks after the injection, and a blood glucose level > 16.7 × 10^−3^ m was defined as a successful diabetic rat model. Sixty diabetic rats were used to evaluate the effects of PARP1@PLS‐PT100 nanospheres on wound repair. Full‐thickness cutaneous wounds (*φ* 20 mm) were created on the backs of rats, and a plastic loop (*φ* 20 mm) was sutured to the skin around the edges of the wound. All rats were randomly divided into five groups, and the groups were treated with PLS (PLS), PLS encapsulating the PARP1 plasmid (PARP1@PLS), PLS scion grafted PT100 (PLS‐PT100), PARP1 plasmid transfected with liposomes (PARP1), or PLS nanosphere scion grafted with PT100 and encapsulating the PARP1 plasmid (PARP1@PLS‐PT100). The rats were fed an HF diet in a specific pathogen‐free (SPF) animal room. Wound closure was captured with a digital camera on days 0, 3, 7, 12, and 21 to observe wound repair progression. After 12 and 21 days, rats were sacrificed, and the wound tissues were collected for histological analysis.

### Wound Healing Rate Assay

Wound closure progression was recorded with a digital camera on days 0, 3, 7, 12, and 21 after surgery. The wound closure simulation plots were created from the digital images of healing wounds captured on days 0, 3, 7, 12, and 21. The area of wound healing was calculated with ImageJ software. From the information, the wound closure rates were calculated using the following formula: wound closure rates = (wound area on day 0− wound area at a certain time point)/wound area on day 0.^[^ ^3^ ^]^

### Histology of the Wound Tissue

For the histological assessment of the wound, wound tissues were fixed and embedded in paraffin. Fixed wound tissue sections were stained with H&E solutions, Masson's trichrome solutions, and Sirius solutions according to the manufacturer's instructions.^[^ ^60^ ^]^ The images of stained slides were then obtained under a Leica microscope.

### Immunohistochemistry

Ki67 immunostaining was performed to evaluate the proliferative ability of the impaired tissue. The wound tissue was fixed with 4% paraformaldehyde fixation solution for 24 h at RT and cut into sections. Immunocytochemistry was performed on the paraffin sections, and the sections were blocked with immunostaining blocking solution (Biyuntian, Shanghai, China) at RT for 30 min and then incubated with an anti‐Ki67 antibody (1:200, Abcam, Cambridge, UK) at 4 °C overnight. Next, the sections were rinsed and incubated with an HRP‐conjugated anti‐rabbit IgG (1:600, Servicebio, Wuhan, China), followed by diaminobenzidine (DAB, Servicebio, Wuhan, China) and weak counterstaining with diluted hematoxylin (Servicebio, Wuhan, China) for 30 seconds. Images of immunohistochemical staining in the sections were acquired using a Leica microscope.

### Statistical Analysis

Statistical analyses were performed as described in the figure legend for each experiment. Statistical significance was determined using one‐ and two‐way ANOVA and Student's *t*‐tests with GraphPad Prism 8 software, as indicated. A *p*‐value less than 0.05 was considered significant and indicated with asterisks: *p <0.05, ns:*p* > 0.05.

## Conflict of Interest

The authors declare no conflict of interest.

## Supporting information

Supporting Information

## Data Availability

Research data are not shared.

## References

[1] D. G. Armstrong , A. J. M. Boulton , S. A. Bus , N. Engl. J. Med. 2017, 376, 2367. 28614678 10.1056/NEJMra1615439

[2] V. Falanga , Lance 2005, 366, 1736. 10.1016/S0140-6736(05)67700-8 16291068

[3] R. A. Dorschner , J. Lee , O. Cohen , T. Costantini , A. Baird , B. P. Eliceiri , Sci. Adv. 2020, 6, eaay0518. 32195341 10.1126/sciadv.aay0518 PMC7065879

[4] S. Wong , M. Demers , K. Martinod , M. Gallant , Y. Wang , A. Goldfine , C. Kahn , D. Wagner , Nat. Med. 2015, 21, 815. 26076037 10.1038/nm.3887 PMC4631120

[5] P. Y. Wu , C. C. Huang , Y. Chu , Y. H. Huang , P. Lin , Y. H. Liu , K. C. Wen , C. Y. Lin , M. C. Hsu , H. M. Chiang , Int. J. Mol. Sci. 2017, 18. 10.3390/ijms18040782. PMC5412366 28387707

[6] T. S. Chikenji , Y. Saito , N. Konari , M. Nakano , Y. Mizue , M. Otani , M. Fujimiya , EBioMedicine 2019, 44, 86. 31129096 10.1016/j.ebiom.2019.05.012 PMC6604166

[7] L. Lau , A. Porciuncula , A. Yu , Y. Iwakura , G. David , Mol. Cell. Biol. 2019, 39. 10.1128/mcb.00586-18. PMC6549465 30988157

[8] M. Oh , J. Lee , Y. J. Kim , W. J. Rhee , J. H. Park , Int. J. Mol. Sci. 2018, 19. 10.3390/ijms19061715. PMC6032439 29890746

[9] D. Furman , J. Chang , L. Lartigue , C. R. Bolen , F. Haddad , B. Gaudilliere , E. A. Ganio , G. K. Fragiadakis , M. H. Spitzer , I. Douchet , S. Daburon , J. F. Moreau , G. P. Nolan , P. Blanco , J. Déchanet‐Merville , C. L. Dekker , V. Jojic , C. J. Kuo , M. M. Davis , B. Faustin , Nat. Med. 2017, 23, 174. 28092664 10.1038/nm.4267 PMC5320935

[10] H. Wilkinson , M. Hardman , Front. Cell Dev. Biol. 2020, 8, 773. 32850866 10.3389/fcell.2020.00773 PMC7431694

[11] A. Wang , O. Dreesen , Front. Genet. 2018, 9, 247. 30190724 10.3389/fgene.2018.00247 PMC6115505

[12] C. Amor , J. Feucht , J. Leibold , Y. Ho , C. Zhu , D. Alonso‐Curbelo , J. Mansilla‐Soto , J. Boyer , X. Li , T. Giavridis , A. Kulick , S. Houlihan , E. Peerschke , S. Friedman , V. Ponomarev , A. Piersigilli , M. Sadelain , S. Lowe , Nature 2020, 583, 127. 32555459 10.1038/s41586-020-2403-9 PMC7583560

[13] J. Farr , M. Xu , M. Weivoda , D. Monroe , D. Fraser , J. Onken , B. Negley , J. Sfeir , M. Ogrodnik , C. Hachfeld , N. LeBrasseur , M. Drake , R. Pignolo , T. Pirtskhalava , T. Tchkonia , M. Oursler , J. Kirkland , S. Khosla , Nat. Med. 2017, 23, 1072. 28825716 10.1038/nm.4385 PMC5657592

[14] D. Y. Zhang , Z. Y. Pan , X. K. Yu , Y. F. Chen , C. H. Gao , Y. T. Yang , X. F. Jiang , N. Li , J. P. Pan , J. Immunol. Res. 2019, 2019, 4657928. 31930149 10.1155/2019/4657928 PMC6942849

[15] A. Kowald , J. F. Passos , T. B. L. Kirkwood , Aging Cell 2020, 19, 13270. 10.1111/acel.13270 PMC7744960 33166065

[16] J. Zhang , J. Sun , Q. Zheng , X. Hu , Z. Wang , Z. Liang , K. Li , J. Song , T. Ding , X. Shen , J. Zhang , L. Qiao , J. Cell. Mol. Med. 2020, 24, 476. 31667932 10.1111/jcmm.14756 PMC6933332

[17] D. Chen , J. Xie , R. Fiskesund , W. Dong , X. Liang , J. Lv , X. Jin , J. Liu , S. Mo , T. Zhang , F. Cheng , Y. Zhou , H. Zhang , K. Tang , J. Ma , Y. Liu , B. Huang , Nat. Commun. 2018, 9, 873. 29491374 10.1038/s41467-018-03225-9 PMC5830447

[18] A. Sindrilaru , T. Peters , S. Wieschalka , C. Baican , A. Baican , H. Peter , A. Hainzl , S. Schatz , Y. Qi , A. Schlecht , J. M. Weiss , M. Wlaschek , C. Sunderkötter , K. Scharffetter‐Kochanek , J. Clin. Invest. 2011, 121, 985. 21317534 10.1172/JCI44490 PMC3049372

[19] A. J. Covarrubias , A. Kale , R. Perrone , J. A. Lopez‐Dominguez , A. O. Pisco , H. G. Kasler , M. S. Schmidt , I. Heckenbach , R. Kwok , C. D. Wiley , H. S. Wong , E. Gibbs , S. S. Iyer , N. Basisty , Q. Wu , I. J. Kim , E. Silva , K. Vitangcol , K. O. Shin , Y. M. Lee , R. Riley , I. Ben‐Sahra , M. Ott , B. Schilling , M. Scheibye‐Knudsen , K. Ishihara , S. R. Quake , J. Newman , C. Brenner , J. Campisi , E. Verdin , Nat. Metab. 2020, 2, 1265. 33199924 10.1038/s42255-020-00305-3 PMC7908681

[20] Y. Cai , H. Zhou , Y. Zhu , Q. Sun , Y. Ji , A. Xue , Y. Wang , W. Chen , X. Yu , L. Wang , H. Chen , C. Li , T. Luo , H. Deng , Cell Res. 2020, 30, 574. 32341413 10.1038/s41422-020-0314-9 PMC7184167

[21] M. Wakita , A. Takahashi , O. Sano , T. M. Loo , Y. Imai , M. Narukawa , H. Iwata , T. Matsudaira , S. Kawamoto , N. Ohtani , T. Yoshimori , E. Hara , Nat. Commun. 2020, 11, 1935. 32321921 10.1038/s41467-020-15719-6 PMC7176673

[22] E. Dookun , A. Walaszczyk , R. Redgrave , P. Palmowski , S. Tual‐Chalot , A. Suwana , J. Chapman , E. Jirkovsky , L. Donastorg Sosa , E. Gill , O. Yausep , Y. Santin , J. Mialet‐Perez , W. Andrew Owens , D. Grieve , I. Spyridopoulos , M. Taggart , H. Arthur , J. Passos , G. Richardson , Aging Cell 2020, 19, 13249. 10.1111/acel.13249 PMC7576252 32996233

[23] H. Salmonowicz , J. F. Passos , Aging Cell 2017, 16, 432. 28185406 10.1111/acel.12580 PMC5418201

[24] M. De Cecco , T. Ito , A. P. Petrashen , A. E. Elias , N. J. Skvir , S. W. Criscione , A. Caligiana , G. Brocculi , E. M. Adney , J. D. Boeke , O. Le , C. Beauséjour , J. Ambati , K. Ambati , M. Simon , A. Seluanov , V. Gorbunova , P. E. Slagboom , S. L. Helfand , N. Neretti , J. M. Sedivy , Nature 2019, 566, 73. 30728521 10.1038/s41586-018-0784-9 PMC6519963

[25] J. Iske , M. Seyda , T. Heinbokel , R. Maenosono , K. Minami , Y. Nian , M. Quante , C. S. Falk , H. Azuma , F. Martin , J. F. Passos , C. U. Niemann , T. Tchkonia , J. L. Kirkland , A. Elkhal , S. G. Tullius , Nat. Commun. 2020, 11, 4289. 32855397 10.1038/s41467-020-18039-x PMC7453018

[26] P. C. Wu , Q. Wang , Z. M. Dong , E. Chu , R. S. Roberson , I. C. Ivanova , D. Y. Wu , Cell Death Dis. 2010, 1, 70. 10.1038/cddis.2010.47 PMC3032339 21364674

[27] M. Ge , L. Hu , H. Ao , M. Zi , Q. Kong , Y. He , Mech. Agein Dev. 2021, 195, 111468. 10.1016/j.mad.2021.111468 33741395

[28] N. Schmid , F. Flenkenthaler , J. B. Stöckl , K.‐G. Dietrich , F. M. Köhn , J. U. Schwarzer , L. Kunz , M. Luckner , G. Wanner , G. J. Arnold , T. Fröhlich , A. Mayerhofer , Sci. Rep. 2019, 9, 15052. 31636313 10.1038/s41598-019-51380-w PMC6803627

[29] S. Elouej , K. Harhouri , M. L.e Mao , G. Baujat , S. Nampoothiri , H. Kayserili , N. Menabawy , L. Selim , A. Paneque , C. Kubisch , D. Lessel , R. Rubinsztajn , C. Charar , C. Bartoli , C. Airault , J. Deleuze , A. Rötig , P. Bauer , C. Pereira , A. Loh , N. Escande‐Beillard , A. Muchir , L. Martino , Y. Gruenbaum , S. Lee , P. Manivet , G. Lenaers , B. Reversade , N. Lévy , A. De Sandre‐Giovannoli , Nat. Commun. 2020, 11, 4589. 32917887 10.1038/s41467-020-18146-9 PMC7486921

[30] A. Mongelli , S. Atlante , V. Barbi , T. Bachetti , F. Martelli , A. Farsetti , C. Gaetano , Int. J. Mol. Sci. 2020, 21. 10.3390/ijms21217984. PMC7014325 31941147

[31] N. Mabrouk , S. Ghione , V. Laurens , S. Plenchette , A. Bettaieb , C. Paul , Cancers 2020, 12. 10.3390/cancers12051145. PMC7281185 32370259

[32] Z. Ma , J. Li , K. Lin , M. Ramachandran , D. Zhang , M. Showalter , C. De Souza , A. Lindstrom , L. N. Solano , B. Jia , S. Urayama , Y. Duan , O. Fiehn , T. Y. Lin , M. Li , Y. Li , Nat. Commun. 2020, 11, 4615. 32934241 10.1038/s41467-020-18399-4 PMC7493904

[33] Y. Wu , Z. Song , H. Wang , H. Han , Nat. Commun. 2019, 10, 4464. 31578336 10.1038/s41467-019-12233-2 PMC6775118

[34] K. Kim , J. Noh , M. Bodogai , J. Martindale , X. Yang , F. Indig , S. Basu , K. Ohnuma , C. Morimoto , P. Johnson , A. Biragyn , K. Abdelmohsen , M. Gorospe , Genes Dev. 2017, 31, 1529. 28877934 10.1101/gad.302570.117 PMC5630018

[35] G. Casella , R. Munk , K. Kim , Y. Piao , S. De , K. Abdelmohsen , M. Gorospe , Nucleic Acids Res. 2019, 47, 11476. 31612919 10.1093/nar/gkz879 PMC6868356

[36] M. Mashimo , M. Onishi , A. Uno , A. Tanimichi , A. Nobeyama , M. Mori , S. Yamada , S. Negi , X. Bu , J. Kato , J. Moss , N. Sanada , R. Kizu , T. Fujii , J. Biol. Chem. 2020, 296, 100046. 33168626 10.1074/jbc.RA120.014479 PMC7948984

[37] A. Joshi , R. Iyengar , J. Joo , X. Li‐Harms , C. Wright , R. Marino , B. Winborn , A. Phillips , J. Temirov , S. Sciarretta , R. Kriwacki , J. Peng , A. Shelat , M. Kundu , Cell Death Differ. 2016, 23, 216. 26138443 10.1038/cdd.2015.88 PMC4716304

[38] Y. Ying , B. Padanilam , Cell. Mol. Life Sci. 2016, 73, 2309. 27048819 10.1007/s00018-016-2202-5 PMC5490387

[39] P. Mukhopadhyay , B. Horváth , M. Rajesh , Z. Varga , K. Gariani , D. Ryu , Z. Cao , E. Holovac , O. Park , Z. Zhou , M. Xu , W. Wang , G. Godlewski , J. Paloczi , B. Nemeth , Y. Persidsky , L. Liaudet , G. Haskó , P. Bai , A. Boulares , J. Auwerx , B. Gao , P. Pacher , J. Hepatol. 2017, 66, 589. 27984176 10.1016/j.jhep.2016.10.023

[40] Y. Zhu , T. Tchkonia , T. Pirtskhalava , A. Gower , H. Ding , N. Giorgadze , A. Palmer , Y. Ikeno , G. Hubbard , M. Lenburg , S. O'Hara , N. LaRusso , J. Miller , C. Roos , G. Verzosa , N. LeBrasseur , J. Wren , J. Farr , S. Khosla , M. Stout , S. McGowan , H. Fuhrmann‐Stroissnigg , A. Gurkar , J. Zhao , D. Colangelo , A. Dorronsoro , Y. Ling , A. Barghouthy , D. Navarro , T. Sano , P. Robbins , L. Niedernhofer , J. Kirkland , Aging Cell 2015, 14, 644. 25754370 10.1111/acel.12344 PMC4531078

[41] Y. Wu , Y. Quan , Y. Liu , K. Liu , H. Li , Z. Jiang , T. Zhang , H. Lei , K. A. Radek , D. Li , Z. Wang , J. Lu , W. Wang , S. Ji , Z. Xia , Y. Lai , Nat. Commun. 2016, 7, 13393. 27830702 10.1038/ncomms13393 PMC5109591

[42] Z. Yang , R. Huang , B. Zheng , W. Guo , C. Li , W. He , Y. Wei , Y. Du , H. Wang , D. Wu , H. Wang , Adv. Sci. 2021, 8, 2003627. 10.1002/advs.202003627 PMC8061386 33898178

[43] K. Y. Lee , D. J. Mooney , Prog. Polym. Sci. 2012, 37, 106. 22125349 10.1016/j.progpolymsci.2011.06.003 PMC3223967

[44] D. Yoon , Y. Lee , H. Ryu , Y. Jang , K. Lee , Y. Choi , W. Choi , M. Lee , K. Park , K. Park , J. Lee , Acta Biomater. 2016, 38, 59. 27109762 10.1016/j.actbio.2016.04.030

[45] J. Mun , K. Shin , O. Kwon , Y. Lim , D. Oh , Biomaterials 2016, 101, 310. 27315214 10.1016/j.biomaterials.2016.05.057

[46] H. Meany , F. Balis , A. Aikin , P. Whitcomb , R. Murphy , S. Steinberg , B. Widemann , E. Fox , J. Natl. Cancer Inst. 2010, 102, 909. 20460632 10.1093/jnci/djq174 PMC2886096

[47] G. Guan , B. Song , J. Zhang , K. Chen , H. Hu , M. Wang , D. Chen , Pharmaceutics 2019, 11. 10.3390/pharmaceutics11110608. PMC6920835 31766300

[48] J. Gonzalez‐Meljem , S. Haston , G. Carreno , J. Apps , S. Pozzi , C. Stache , G. Kaushal , A. Virasami , L. Panousopoulos , S. Mousavy‐Gharavy , A. Guerrero , M. Rashid , N. Jani , C. Goding , T. Jacques , D. Adams , J. Gil , C. Andoniadou , J. Martinez‐Barbera , Nat. Commun. 2017, 8, 1819. 29180744 10.1038/s41467-017-01992-5 PMC5703905

[49] B. Dyett , H. Yu , J. Strachan , C. Drummond , C. Conn , Nat. Commun. 2019, 10, 4492. 31582802 10.1038/s41467-019-12508-8 PMC6776645

[50] Y. Wang , B. Schulte , A. LaRue , M. Ogawa , D. Zhou , Blood 2006, 107, 358. 16150936 10.1182/blood-2005-04-1418 PMC1895367

[51] P. Zhu , C. Zhang , Y. Gao , F. Wu , Y. Zhou , W. Wu , Nat. Commun. 2019, 10, 2568. 31189923 10.1038/s41467-019-10479-4 PMC6561969

[52] F. Chellini , A. Tani , L. Vallone , D. Nosi , P. Pavan , F. Bambi , S. Z. Orlandini , C. Sassoli , Cells 2018, 7. 10.3390/cells7090142. PMC6162453 30235859

[53] B. Shook , R. Wasko , O. Mano , M. Rutenberg‐Schoenberg , M. Rudolph , B. Zirak , G. Rivera‐Gonzalez , F. López‐Giráldez , S. Zarini , A. Rezza , D. Clark , M. Rendl , M. Rosenblum , M. Gerstein , V. Horsley , Cell Stem Cell 2020, 26, 880. 32302523 10.1016/j.stem.2020.03.013 PMC7853423

[54] S. Jimi , M. Kimura , F. De Francesco , M. Riccio , S. Hara , H. Ohjimi , Int. J. Mol. Sci. 2017, 18. 10.3390/ijms18081675. PMC5578065 28767054

[55] S. Elouej , K. Harhouri , M. L.e Mao , G. Baujat , S. Nampoothiri , H. Kayserili , N. A. Menabawy , L. Selim , A. L. Paneque , C. Kubisch , D. Lessel , R. Rubinsztajn , C. Charar , C. Bartoli , C. Airault , J. F. Deleuze , A. Rötig , P. Bauer , C. Pereira , A. Loh , N. Escande‐Beillard , A. Muchir , L. Martino , Y. Gruenbaum , S. H. Lee , P. Manivet , G. Lenaers , B. Reversade , N. Lévy , A. De Sandre‐Giovannoli , Nat. Commun. 2020, 11, 4589. 32917887 10.1038/s41467-020-18146-9 PMC7486921

[56] H. Wu , F. Li , W. Shao , J. Gao , D. Ling , ACS Cent. Sci. 2019, 5, 477. 30937375 10.1021/acscentsci.8b00850 PMC6439452

[57] C. Y. Chen , H. Yin , X. Chen , T. H. Chen , H. M. Liu , S. S. Rao , Y. J. Tan , Y. X. Qian , Y. W. Liu , X. K. Hu , M. J. Luo , Z. X. Wang , Z. Z. Liu , J. Cao , Z. H. He , B. Wu , T. Yue , Y. Y. Wang , K. Xia , Z. W. Luo , Y. Wang , W. Y. Situ , W. E. Liu , S. Y. Tang , H. Xie , Sci. Adv. 2020, 6. 10.1126/sciadv.aba0942.

[58] C. Sanhueza , S. Wehinger , J. Castillo Bennett , M. Valenzuela , G. I. Owen , A. F. Quest , Mol. Cancer 2015, 14, 198. 26584646 10.1186/s12943-015-0467-1 PMC4653922

[59] C. Crochemore , C. Fernández‐Molina , B. Montagne , A. Salles , M. Ricchetti , Nat. Commun. 2019, 10, 5576. 31811121 10.1038/s41467-019-13314-y PMC6898346

[60] A. Georgilis , S. Klotz , C. Hanley , N. Herranz , B. Weirich , B. Morancho , A. Leote , L. D'Artista , S. Gallage , M. Seehawer , T. Carroll , G. Dharmalingam , K. Wee , M. Mellone , J. Pombo , D. Heide , E. Guccione , J. Arribas , N. Barbosa‐Morais , M. Heikenwalder , G. Thomas , L. Zender , J. Gil , Cancer Cell 2018, 34, 85. 29990503 10.1016/j.ccell.2018.06.007 PMC6048363

[61] J. Han , M. Yu , Y. Bai , J. Yu , F. Jin , C. Li , R. Zeng , J. Peng , A. Li , X. Song , H. Li , D. Wu , L. Li , Cancer Cell 2020, 38, 844. 33186520 10.1016/j.ccell.2020.10.009 PMC8455074

[62] G. Orlando , S. Khoronenkova , I. Dianova , J. Parsons , G. Dianov , Nucleic Acids Res. 2014, 42, 2320. 24293653 10.1093/nar/gkt1185 PMC3936746

[63] C. Dinhof , C. Pirker , P. Kroiss , D. Kirchhofer , L. Gabler , J. Gojo , D. Lötsch‐Gojo , M. Stojanovic , G. Timelthaler , F. Ferk , S. Knasmüller , J. Reisecker , S. Spiegl‐Kreinecker , P. Birner , M. Preusser , W. Berger , Cancers 2020, 12. 10.3390/cancers12113205. PMC7693367 33143299

[64] S. D. Stojanović , J. Fiedler , J. Bauersachs , T. Thum , D. G. Sedding , Eur. Heart J. 2020, 41, 2983. 31898722 10.1093/eurheartj/ehz919 PMC7453834

[65] Y. Ovadya , T. Landsberger , H. Leins , E. Vadai , H. Gal , A. Biran , R. Yosef , A. Sagiv , A. Agrawal , A. Shapira , J. Windheim , M. Tsoory , R. Schirmbeck , I. Amit , H. Geiger , V. Krizhanovsky , Nat. Commun. 2018, 9, 5435. 30575733 10.1038/s41467-018-07825-3 PMC6303397

[66] L. Vi , G. Baht , E. Soderblom , H. Whetstone , Q. Wei , B. Furman , V. Puviindran , P. Nadesan , M. Foster , R. Poon , J. White , Y. Yahara , A. Ng , T. Barrientos , M. Grynpas , M. Mosely , B. Alman , Nat. Commun. 2018, 9, 5191. 30518764 10.1038/s41467-018-07666-0 PMC6281653

[67] E. Y. Shin , J. H. Park , S. T. You , C. S. Lee , S. Y. Won , J. J. Park , H. B. Kim , J. Shim , N. K. Soung , O. J. Lee , M. A. Schwartz , E. G. Kim , Sci. Adv. 2020, 6, eaay3909. 32494696 10.1126/sciadv.aay3909 PMC7202880

[68] T. Chen , T. Wen , N. Dai , S. Hsu , Biomaterials 2021, 269, 120608. 33388690 10.1016/j.biomaterials.2020.120608

[69] B. I. Pereira , O. P. Devine , M. Vukmanovic‐Stejic , E. S. Chambers , P. Subramanian , N. Patel , A. Virasami , N. J. Sebire , V. Kinsler , A. Valdovinos , C. J. LeSaux , J. F. Passos , A. Antoniou , M. H. A. Rustin , J. Campisi , A. N. Akbar , Nat. Commun. 2019, 10, 2387. 31160572 10.1038/s41467-019-10335-5 PMC6547655

[70] J. Cohen , C. Torres , Aging Cell 2019, 18, 12937. 10.1111/acel.12937 PMC6516680 30815970

[71] K. Suzuki , M. Yamamoto , R. Hernandez‐Benitez , Z. Li , C. Wei , R. D. Soligalla , E. Aizawa , F. Hatanaka , M. Kurita , P. Reddy , A. Ocampo , T. Hishida , M. Sakurai , A. N. Nemeth , E. Nuñez Delicado , J. M. Campistol , P. Magistretti , P. Guillen , C. Rodriguez Esteban , J. Gong , Y. Yuan , Y. Gu , G. H. Liu , C. López‐Otín , J. Wu , K. Zhang , J. C. Izpisua Belmonte , Cell Res. 2019, 29, 804. 31444470 10.1038/s41422-019-0213-0 PMC6796851

[72] R. Assi , T. R. Foster , H. He , K. Stamati , H. Bai , Y. Huang , F. Hyder , D. Rothman , C. Shu , S. Homer‐Vanniasinkam , U. Cheema , A. Dardik , Regener. Med. 2016, 11, 245. 10.2217/rme-2015-0045 PMC4976993 26986810

