# Supplementary material for: Precise Diabetic Wound Therapy: PLS Nanospheres Eliminate Senescent Cells via DPP4 Targeting and PARP1 Activation
Source: Adv Sci (Weinh). 2021 Nov 5;9(1):2104128. doi: 10.1002/advs.202104128 (PMC8728814; doi:10.1002/advs.202104128)
Supplement: Supplementary file 1 — Supporting Information [file ADVS-9-2104128-s001.pdf]

ADVANCED SCIENCE
Open Access

Supporting Information

for *Adv. Sci.*, DOI: 10.1002/advs.202104128

Precise diabetic wound therapy: PLS nanospheres eliminate senescent cells via DPP4 targeting and PARP1 activation

Renliang Zhao, Xiangyun Jin, Ang Li, Bitong Xu, Yifan Shen, Wei Wang, Jinghuan Huang, Yadong Zhang*, Xiaolin Li**

Precise diabetic wound therapy: PLS nanospheres eliminate senescent cells via DPP4 targeting and PARP1 activation

Renliang Zhao[1,#], Xiangyun Jin[3,#], Ang Li[1,#], Bitong Xu[2], Yifan Shen[1], Wei Wang[1], Jinghuan Huang[1,], Yadong Zhang [2,]*, Xiaolin Li[1,]**

1. Department of Orthopedic Surgery, and Shanghai Institute of Microsurgery on Extremities, Shanghai Jiao Tong University Affiliated Sixth People's Hospital, Shanghai 200233, China.
2. Department of spine,Center for orthopaedic surgery,The third affiliated hospital of southern medical university 510515,Guangzhou,China
3. Department of Orthopedic Trauma, Renji Hospital, School of Medicine, Shanghai Jiao Tong University, 200127 Shanghai, PR China

∗ Corresponding authors:
Jinghuan Huang Email: 371222550@sjtu.edu.cn
Yadong Zhang Email: zhangyadong6@126.com
Xiaolin Li Email: lixiaolin@sjtu.edu.cn

[#]These authors contributed equally to the work.

Additional experimental methods

PLS-PT100/pDNA nanosphere hydrogel preparation and characterization

pDNA-PARP1 was encapsulated into PLS-PT100 nanospheres. According to the instructions (Sangon Biotech, Shanghai, China), pDNA was obtained from *E. coli* growing in tryptone media and purified. A series of concentrations of PLS-PT100 was dissolved in ddH_2O and sterilized through a 0.22 μm filter to obtain the PLS-PT100/pDNA nanospheres. The PLS-PT100 solution was stirred and mixed with the pDNA solution. The capability of PLS-PT100 nanospheres to encapsulate pDNA was tested by performing agarose gel electrophoresis88. A 10 μl volume was prepared with 8 μl of PLS-PT100/DNA nanospheres at various ratios and 2 μl of diluted loading buffer, which was added to the agarose gel (1%) with EB (0.5 μl/ml) and Tris-acetic acid-EDTA (TAE) buffer. Electrophoretic mobility of the samples was measured by electrophoresis at 100 V for 45 min at RT. A digital camera was used to capture the images of the pDNA bands under a UV illuminator[1].

Transfection of the PARP1 plasmid

The PARP1 gene was used to compare the transfection efficiency between the nanospheres and Lipofectamine in the SFs. Briefly, cells were seeded in plates at a density of $2.5x10^5$ cells/well and cultured for 24 hours. Before transfection, the cells were washed once with Opti-MEM reduced serum medium (Thermo Fisher), and two transfection methods were used to transfer the plasmid, which have been reconstructed as the PARP1-GFP fusion. For Lipofectamine-mediated lipofection, 2.5 μg of pDNA and 5 μl of the Lipofectamine complex were mixed, added to the cell suspension and incubated for 12 h after transfection. The plates were washed with PBS three times, and fresh complete medium was added. For the nanosphere method, the cells were washed once with Opti-MEM reduced serum medium, and 100 μg/ml nanospheres (containing 2.5 μg of pDNA) were added to the plates. All cells were incubated in an incubator for 12 h and then changed to fresh complete medium. Thereafter, the cells were cultured for 24 h and resuspended for transgene expression investigation. The transfection efficiency was evaluated with GFP fluorescence, Western blot, and RT-PCR experimental techniques[2].

β-Galactosidase staining

Frozen sections were equilibrated at RT for 10 min and then fixed with β-galactosidase fixation solution for 30 min at RT. The sections were blocked and incubated with X-gal working solution (Solarbio, Beijing, China) at 37° C for at least 24 h in the dark without CO_2. The sections were washed with PBS three times and then stained with nuclear fast red solution (Servicebio, Wuhan, China). Images of β-galactosidase staining were obtained using a Leica microscope[3].

Immunofluorescence staining:

Paraffin sections were prepared using the same method as described for immunohistochemistry[4]. The sections were incubated overnight with an anti-CD206 primary antibody (1:1000 Abcam, Cambridge, UK), rinsed and incubated with Cy5 anti-rabbit

IgG (1:400, Servicebio, Wuhan, China) in the dark for 1 h. DAPI was used for nuclear labeling and incubated with the sections for 10 min before sections were sealed with immunostaining sealing solution (Servicebio, Wuhan, China). Images were acquired using a Leica microscope. TUNEL staining was performed to evaluate the apoptosis of SFs. The paraffin sections were incubated with TUNEL solutions according to the instructions (Servicebio, Wuhan, China) and restained with DAPI. Images of TUNEL staining were obtained with a digital camera attached to a Leica microscope[5].

Immunofluorescence staining of P16^{INK4a} and α-SMA

For immunofluorescence staining[6], SFs were seeded in plates at a density of 2.8x10^4 cells/cm^2 to evaluate the expression of P16^{INK4a}. Subsequently, the supernatant was collected to confirm the effect of SFs on normal fibroblasts. A total of 2.8x10^4 SFs/cm^2 were incubated in the plates for 24 h, and then, the medium was replaced with 200 μl fresh complete medium containing 100 μg/ml nanospheres. After 3 days, the supernatant was collected and incubated with HFF-1 cells at a density of 2.8x10^4 cells/cm^2 for 3 days. The HFF-1 cells were rinsed three times, fixed with 4% paraformaldehyde, permeabilized with 0.2% Triton-X100 for 30 minutes, rinsed three times with PBS, and then stained with primary antibodies against α-SMA (Abcam, Cambridge, UK) (1:200 dilution) in PBS supplemented with 2% goat serum. Additionally, SFs were stained with a P16^{INK4a} (Abcam, Cambridge, UK) antibody using the same method. RBITC- and FITC-labeled antibodies (Yeasen, China) were used as secondary antibodies. The cytoskeleton was visualized with rhodamine-conjugated phalloidin (RBITC-phalloidin, Yeasen, China, dilution 1:150). In addition, cell nuclei were stained with DAPI (1:200, Solarbio, Beijing, China). The expression of P16^{INK4a} and α-SMA was observed using a Leica immunofluorescence microscope[7].

Inflammation of the SFs

The concentrations of proinflammatory factors and anti-inflammatory factors in the cell supernatant were measured using R&D ELISA kits, including the Human TNF-α Quantikine

ELISA Kit, Human IL-3 Quantikine ELISA Kit, Human IL-6 Quantikine ELISA Kit, Human IL-1α/IL-1F1 Quantikine ELISA Kit, Human IFN-γ Quantikine ELISA Kit, and Human G-CSF Quantikine HS ELISA Kit (HSTA00E) (from R&D Systems, Minneapolis, MN) according to the manufacturer's instructions. The expression of the proinflammatory factors IFN-γ, IL-6, TNF-α, and IL-1 in fibroblasts was detected, and the expression of the anti-inflammatory factors G-CSF and IL-3 was also detected. SFs (2.8x 10^4 cells/cm^2) were seeded in the plates and incubated for 3 days after treatment with 200 μl（100 μg/ml) nanospheres. The supernatant was collected and cultured with HFF-1 cells at a density of 2.8x10^4 cells/cm^2. Then, the cell culture medium was collected and subjected to ELISAs according to the instructions after 48 hours. A multidetection microplate reader (BioTek, USA) was used to detect the absorbance, and the quantitation was performed according to the instructions[8].

RT-PCR

The wound tissue was collected and stored in liquid nitrogen. Total cellular RNA was isolated by grinding the tissue in lysis reagent, and reverse transcription was performed using the Revert First-Strand cDNA Synthesis Kit (EZBioscience, US) with total RNA (1 μg). A reaction volume system (10 μl of SYBR Green Master Mix, 0.8 μl of primers, 2 μl of cDNAs, and 7.2 μl of H_2O) was established with SYBR Green Master Mix (EZBioscience, US) according to the manufacturer's instructions. The internal reference for mRNA was glyceraldehyde 3-phosphate dehydrogenase (GAPDH). The primers are listed in the supplementary table. The 2−ΔΔCT method was used to calculate relative gene expression. The results are presented with three replicates[9].

WB

Western blot was performed as described in previous studies. The wound tissue was harvested on day 12 after surgery and lysed in RIPA buffer. Total proteins were isolated, and BCA protein assay kits were used to detect the protein concentration. Proteins were electrophoretically separated on a 10% SDS-PAGE gel at 110 V for 1 h. The membranes to

which proteins were transferred were blocked with 5% fat-free milk and then sequentially incubated with primary antibodies and secondary antibodies. An enhanced chemiluminescence reagent (Thermo Fisher Scientific) was used for imaging. Primary antibodies against PARP1 and AIF were obtained from Abcam (Cambridge, UK)[9].

Flow cytometry

12 and 21 days after surgery, wound tissue was digested with trypsin for flow cytometry. The cells were suspended in cell staining buffer and incubated with a CD16/32 antibody (Santa Cruz Biotechnology, sc-377009). Then, the cells were stained with a CD206 antibody (Abcam, Cambridge, ab223961) and CCR7 antibody (Abcam, Cambridge, ab185745) for 30 min in the dark. The sample was detected using the CytoFLEX Platform (Abcam, Cambridge, UK) according to the manufacturer's instructions. The flow cytometry analysis of FSP-1 (Biolegend, 1:100) and Ki67 (Biolegend, 1:100) was performed using the same method[10].

RBITC labeling of nanospheres

PLL labeled with RBITC was purchased from Chinese Peptide Inc. (Hangzhou, China). The labeled PLL was used to synthesize PLS-PT100 nanospheres as described above. HFF-1 cells and SFs(5.6×10^4 cells/well) were cultured in 24-well plates. Cells were attached to plates 24 hours after incubation, and 500 μl of culture medium were replaced with 100 μg/ml rhodamine B isothiocyanate (RBITC)-labeled nanosphere-containing medium for 24 hours. The group were as devided into three: PLS nanospheres were added in to SFs (PLS@SFs), PLS-PT100 nanospheres were added into HFF-1 (PLS-PT100@HFF-1), and SFs (PLS-PT100@SFs), respectively. Then, the medium was removed from the plates, and the plates were rinsed with PBS three times. Fixation was performed for 30 min at room temperature (RT) with 4% paraformaldehyde solution, images were observed using a Leica immunofluorescence microscope.

Rat collagen type I and III ELISA assay

Wound tissue were collected at post-wounding 12 and 21 days respectively. 1g wound tissue and 5ml PBS were added into tissue homogenizer on ice. The tissue were then centrifuged at 5000 g/min and supernatant were collected to ELISA assay according to the instructions. An rat collagen type I and III ELISA kit (Cloud-clone Corp. SEA571Ra and SEA176Ra).

Supplementary figures

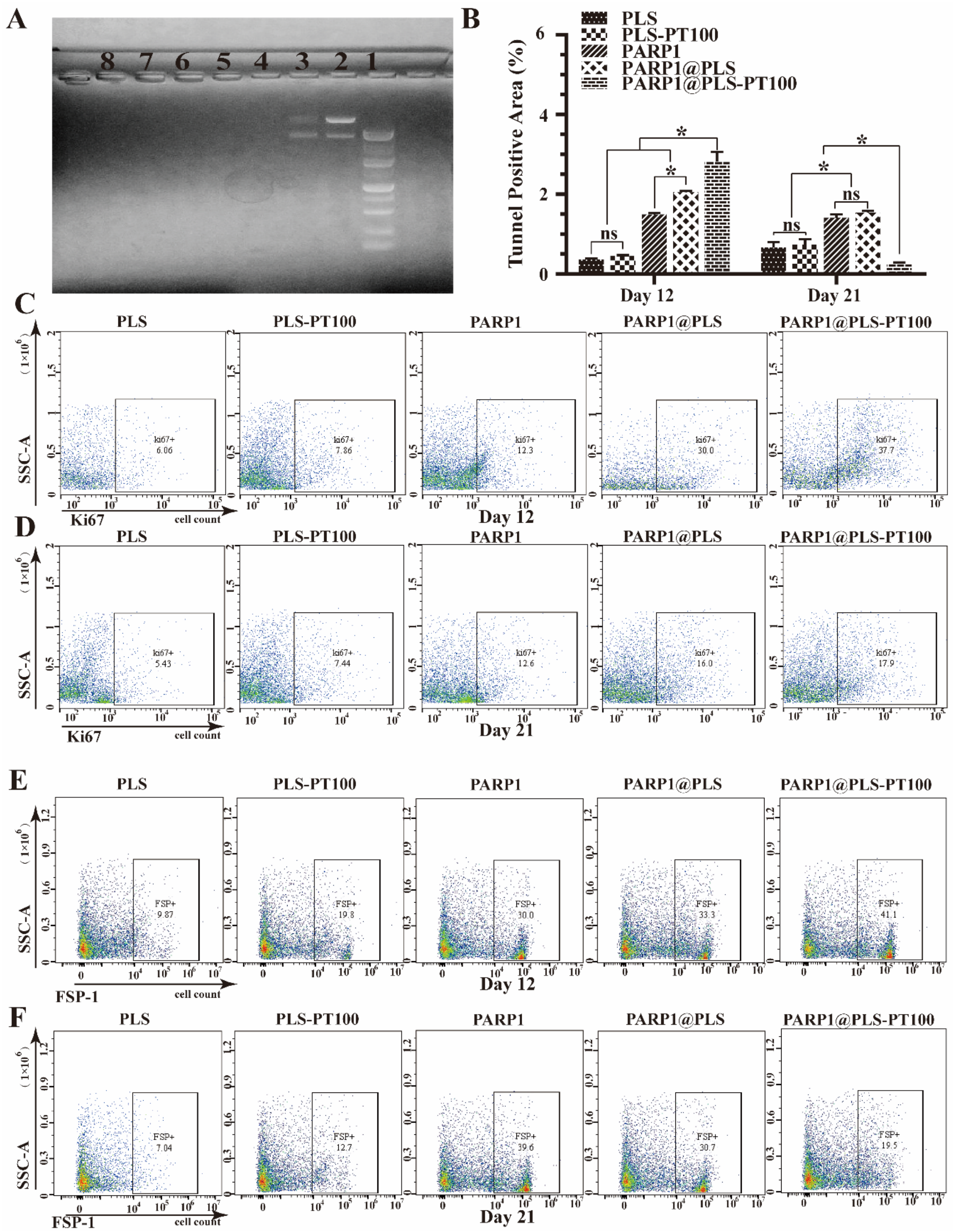

Figure S1. The loading ability of nanospheres was evaluated with nucleic acid electrophoresis (A); 1 represents the marker, 2 represents lipofectamine, and 3–8 represent a series pDNA concentrations (50 µg, 40 µg, 30 µg, 20 µg, 10 µg, and 5 µg). The loading amount was determined based on the electrophoresis results, and 400 µg nanospheres containing 40 µg plasmid was considered an appropriate loading amount. The TUNEL-positive cell area of the wound tissue at day 12 and day 21 after the treatment (B). Flow cytometry analysis of Ki67 expression in wound tissue at day 12 and day 21 (C and D). Flow cytometry analysis of fibroblast-specific protein-1 (FSP-1) expression in wound tissue at day 12 and day 21 (E and F). (n=3 per group; * $P<0.05$, ns, $P>0.05$).

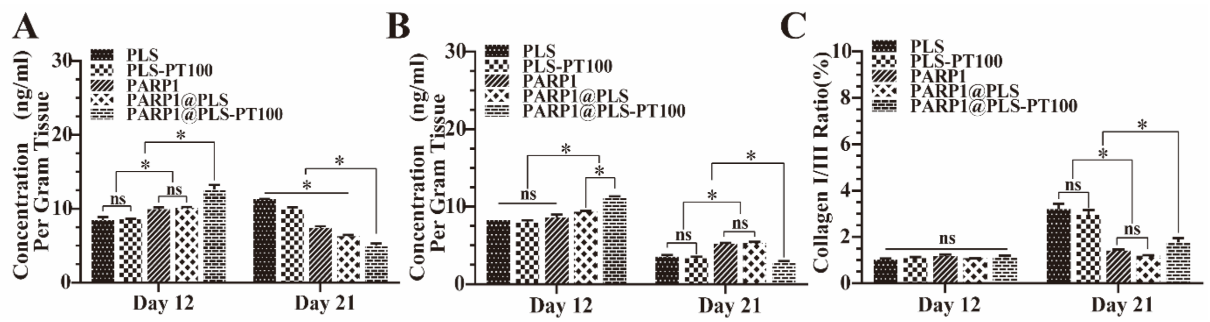

Figure S2. Rat collagen type I and III ELISA of wound tissue at day 12 and day 21 postwounding (A and B). The collagen I/III ratio was calculated according to the results of the ELISA (C). (n=3 per group; * P<0.05, ns, P>0.05).

[1] R. Shi, W. Lian, S. Han, C. Cao, Y. Jin, Y. Yuan, H. Zhao, M. Li, *Gene therapy* **2018**, *25* (6), 425.

[2] D. Niu, Z. Liu, Y. Li, X. Luo, J. Zhang, J. Gong, J. Shi, *Advanced materials (Deerfield Beach, Fla.)* **2014**, *26* (29), 4947.

[3] D. Muñoz-Espín, M. Rovira, I. Galiana, C. Giménez, B. Lozano-Torres, M. Paez-Ribes, S. Llanos, S. Chaib, M. Muñoz-Martín, A. C. Ucero, G. Garaulet, F. Mulero, S. G. Dann, T. VanArsdale, D. J. Shields, A. Bernardos, J. R. Murguía, R. Martínez-Máñez, M. Serrano, *EMBO Mol Med* **2018**, *10* (9).

[4] S. Paul, S. Chhatar, A. Mishra, G. Lal, *Journal for immunotherapy of cancer* **2019**, *7* (1), 208.

[5] B. W. Sperry, B. A. Reyes, A. Ikram, J. P. Donnelly, D. Phelan, W. A. Jaber, D. Shapiro, P. J. Evans, S. Maschke, S. E. Kilpatrick, C. D. Tan, E. R. Rodriguez, C. Monteiro, W. H. W. Tang, J. W. Kelly, W. H. Seitz, Jr., M. Hanna, *Journal of the American College of Cardiology* **2018**, *72* (17), 2040.

[6] L. Engelmann, J. Thierauf, N. Koerich Laureano, H. J. Stark, E. S. Prigge, D. Horn, K. Freier, N. Grabe, C. Rong, P. Federspil, K. Zaoui, P. K. Plinkert, N. Rotter, M. von Knebel Doeberitz, J. Hess, A. Affolter, *Cancers (Basel)* **2020**, *12* (8).

[7] Y. Sun, Z. Yang, B. Zheng, X. H. Zhang, M. L. Zhang, X. S. Zhao, H. Y. Zhao, T. Suzuki, J. K. Wen, *Circulation research* **2017**, *121* (6), 628.

[8] J. Shin, J. H. Kim, E. K. Kim, *Journal of the European Academy of Dermatology and Venereology : JEADV* **2012**, *26* (12), 1577.

[9] D. Zhou, T. Liu, S. Wang, W. He, W. Qian, G. Luo, *Front Physiol* **2020**, *11*, 545008.

[10] J. Hu, T. Wei, H. Zhao, M. Chen, Y. Tan, Z. Ji, Q. Jin, J. Shen, Y. Han, N. Yang, L. Chen, Z. Xiao, H. Zhang, Z. Liu, Q. Chen, *Matter* **2021**, *4* (9), 2985.
